# Research Letter: Unmet Need for Hypertension Treatment in India: Evidence from Hypertension Drugs Market Data

**DOI:** 10.5334/gh.973

**Published:** 2021-04-23

**Authors:** Anupam Khungar Pathni, Swagata Kumar Sahoo, Andrew E. Moran, Jennifer Cohn, Sanchit Bhatia, Nilesh Maheshwari, Bhawna Sharma

**Affiliations:** 1 Resolve to Save Lives, an initiative of Vital Strategies, New York, US; 2 Columbia University Irving Medical Center, New York, US; 3 Division of Infectious Diseases, University of Pennsylvania School of Medicine, Philadelphia, US; 4 Department of Analytics, IQVIA Consulting and Information Services, New Delhi, IN

**Keywords:** hypertension treatment, hypertension drugs, public health

In India, more than 200 million adults are estimated to have high blood pressure [1]. The private sector is the dominant healthcare provider and provides 70% of total outpatient care in the country [2]. Antihypertensive drug sales data can provide empirical evidence on the number of patients receiving hypertension treatment from the private pharmaceutical market. Comparing hypertension prevalence to pharmaceutical sales data, we estimated the unmet need for hypertension treatment in India overall and within five selected states that were the foci of a national hypertension control program (Kerala, Madhya Pradesh, Maharashtra, Punjab, Telangana).

Market sales of all antihypertensive formulations in 2018 were analysed from a large, nationally representative dataset that captured 95% of the total private pharmaceuticals market sales and was collected by IQVIA (formerly Quintiles and IMS Health, Inc.) [3]. The data was thereafter adjusted to represent the total private pharmaceutical market at the national and state level. Total adult population (18+ years) was estimated using 2011 census data adjusted to 2018. Prevalence of hypertension was based on two large household surveys carried out between 2012 and 2014 [4]. In order to standardise across varying strengths of formulations, sales of each antihypertensive drug molecule were converted to defined daily dose (DDD) using standard World Health Organization (WHO) DDD methodology [5]. The DDD is the assumed average daily maintenance dose for the main indication of a drug in adults. For the few single molecule drugs where WHO-DDD was not available, the most frequently sold strength was considered the DDD. Studies have shown that, with the exception of beta blockers, for all classes of antihypertensive drugs the prescribed daily dose (PDD) is consistently greater than DDD, and almost double for angiotensin converting enzyme (ACE) inhibitors and angiotensin receptor blockers (ARBs) [6,7]. Using the IQVIA data, the estimated number of patients on antihypertensive treatment were calculated based on a conservative prescription scenario of (PDD:DDD = 1.2) for single molecule drugs and (PDD:DDD = 1) for fixed-dose combinations (FDC) and the unmet need for treatment inferred.

Total sales of antihypertensive drugs in the private pharmaceutical market in India in 2018 was 22,175 million pills. Sales of FDCs contributed to 39.1% of the total antihypertensive market by volume. In terms of DDDs, the total sales of antihypertensive drugs in 2018 were 18,987 million, which translated to 236 DDDs per 1000 adults with hypertension per day. Based on the prescription scenario considered, the estimated proportion of adults with hypertension in India receiving antihypertensive drugs from the private pharmaceutical market per day in 2018 was 21.5% (Table 1).

**Table 1:** Analysis of antihypertensive drug sales in the private pharmaceutical market in India and five states in 2018: Defined daily dose (DDD) per 1000 hypertensive adults per day and estimated proportion of adults with hypertension receiving antihypertensive drugs per day.

Indicators		State-level					All-India

		Kerala	Maharashtra	Telangana	Punjab	Madhya Pradesh	

a.	Total anti-hypertensive drug sales (pills in million)*	1800	3669	842	729	805	22,175
b.	Total sales of single molecule drugs in DDDs (in million)*	1058	1630	347	337	332	10,312
c.	Total sales of FDCs in DDDs (in million)*	370	1502	360	299	377	8,675
d.	Prevalence of hypertension among adults (18+ years)	34.5%	24.8%	20%	34.8%	21.9%	25.3%
e.	Estimated number of adults with hypertension in 2018 (in million)	8.9	21.2	5.2	7.4	11.1	220.4
A.	DDDs per 1000 adults with hypertension per day	**441**	**404**	**372**	**234**	**175**	**236**
B.	Proportion of estimated adult hypertensive population receiving anti-hypertensive drugs per day	**38.7%**	**36.9%**	**34.2%**	**21.4%**	**16.1%**	**21.5%**

*Note*: * Volume of sales adjusted for 100% sales against 95% coverage of the data set. Calculations: DDDs per 1000 adults with hypertension per day in 2018 is calculated by dividing total sales in DDDs by the estimated number of adults with hypertension as per the following formula: A = (b+c)/e * (1000/365) Proportion of estimated adult population with hypertension receiving antihypertensive drugs per day in 2018 is calculated using a prescription scenario of (PDD:DDD = 1.2) for single molecule drugs and (PDD:DDD = 1) for fixed-dose combination (FDC) drugs as per the following formula: B = [(b/1.2)+c]/e * (100/365)

Considering public sector contribution of 30% to treatment coverage at the national level, no more than 30.7% of the estimated proportion of adults with hypertension received antihypertensive drugs per day in 2018. However, the unmet need is likely to be higher as a significant proportion of patients seeking care in the public sector may be purchasing their medicines from private pharmacies due to their non-availability at public health facilities. A six-state survey carried out in 2004–05, revealed that the median availability of a basket of 27 essential medicines in public sector outlets was 0–30% [8]. More recently, a study from North India found that the availability of antihypertensive drugs in public health facilities was only 60% [9]. Our study findings align with other studies that have also shown a high unmet need for hypertension treatment in India. A systemic review and meta-analysis revealed that only 25% rural and 38% urban adults with hypertension in India were receiving treatment [10]. A nationally representative household survey conducted in 2015–16 among the 15–49 year age group found that only 13.3% of people with hypertension were on treatment [11]. High unmet need for treatment could be due to a combination of several factors, including but not limited to low population awareness, patient health seeking behaviour, health care accessibility and affordability, physician inertia in treatment initiation and escalation.

State level analysis revealed wide variation in the estimated proportion of hypertensive population receiving antihypertensive drugs from the private pharmaceutical market, the lowest unmet need being in Kerala, while the highest unmet need was in Madhya Pradesh (Table 1). In India, the health systems and public health budgets vary between states. If state-level data on public procurement of antihypertensive drugs is known, the total unmet need can be more specifically quantified for each state; however, this data is currently not available in the public domain.

Our study has some limitations. We considered the total hypertension drug market for our analysis, while several antihypertensive drugs are also indicated for other cardiovascular and non-cardiovascular conditions, hence the unmet need may be higher. Secondly, there may be significant overlap between the private and public sector markets, though we tried to mitigate this scenario by considering up to 30% contribution by the public sector at the national level. Thirdly, our study only estimated the unmet need for treatment and not for blood pressure control. Patients on treatment with uncontrolled blood pressure also qualify as having an unmet need for treatment. Fourthly, although the sources for the IQVIA data are updated annually, there may be gaps in the completeness of the data.

While community-level surveys can provide useful information relating to unmet need for care, they are resource intensive and conducted only at periodic intervals. In case of chronic diseases where the burden of disease is known, drug sales data can be a useful proxy for estimating the number of patients receiving treatment and hence the unmet need.

In conclusion, in a comprehensive analysis of the Indian pharmaceutical, sales we found that only about one-fifth the need for antihypertensive medicine treatment is currently met by the private market. Hypertension is one of the most important risk factors for cardiovascular disease and improving hypertension treatment coverage and control is key to achieving universal health coverage as envisaged under the sustainable development goals. High burden of hypertension along with high unmet need for hypertension treatment in India needs to be addressed by varied interventions at patient, provider, and health systems levels.
